# *Trypanosoma cruzi* Entrance through Systemic or Mucosal Infection Sites Differentially Modulates Regional Immune Response Following Acute Infection in Mice

**DOI:** 10.3389/fimmu.2013.00216

**Published:** 2013-07-26

**Authors:** Juliana de Meis, Juliana Barreto de Albuquerque, Danielle Silva dos Santos, Désio Aurélio Farias-de-Oliveira, Luiz Ricardo Berbert, Vinícius Cotta-de-Almeida, Wilson Savino

**Affiliations:** ^1^Laboratory of Thymus Research, Oswaldo Cruz Institute, Oswaldo Cruz Foundation, Rio de Janeiro, Brazil

**Keywords:** *Trypanosoma cruzi*, lymph nodes, spleen, cytokine, T cell activation

## Abstract

Acute Chagas disease is characterized by a systemic infection that leads to the strong activation of the adaptive immune response. Outbreaks of oral contamination by the infective protozoan *Trypanosoma cruzi* are frequent in Brazil and other Latin American countries, and an increased severity of clinical manifestations and mortality is observed in infected patients. These findings have elicited questions about the specific responses triggered after *T. cruzi* entry via mucosal sites, possibly modulating local immune mechanisms, and further impacting regional and systemic immunity. Here, we provide evidence for the existence of differential lymphoid organ responses in experimental models of acute *T. cruzi* infection.

Chagas disease (American *Trypanosomiasis*) is a neglected tropical illness caused by the hemoflagellate protozoan *Trypanosoma cruzi*. This infection is considered a world-wide health problem with a lack of treatment options due to the absence of a vaccine and global spreading (1, 2). *Trypanosoma cruzi* infection was initially endemic in rural areas of Latin America and, transmitted by contaminated insect vectors of the *Reduviidae* family. Insects become vectors after biting *T. cruzi*-infected animals or humans. The parasite can also be directly transmitted by blood transfusion and organ transplantation as well as orally and congenitally (3, 4). The incubation period (the time between *T. cruzi* exposure and development of symptoms) varies according to the infection route; for example, transmission by vector has an incubation time of 4–15 days. In cases of transfusion the incubation time ranges from 8 to 120 days, whereas in congenital transmission it varies from 3 to 22 days; roughly the same for oral transmission (5, 6). Today, the oral infection is the most common transmission route in the Brazilian Amazon region after the pan-American Health Organization declared the abrogation of vectorial transmission in the area (5). Interestingly, oral transmission has been associated with high mortality and morbidity due to an increased prevalence and severity of cardiac pathology (myocarditis) (7–10).

## *Trypanosoma cruzi* Infection Targets Several Tissues in the Host

During the acute phase of the disease, the parasites highly replicate in tissues (under amastigote form), and infective trypomastigote forms are numerous in the blood. Throughout this phase, *T. cruzi* is able to infect many host tissues, including skeletal muscle, lymphoid and nervous tissues, endocrine glands, and mucose (Table 1).

**Table 1:** **Tissue infectivity of *Trypanosoma cruzi* in mammals**.

Target tissue	Human		Animals	
	Acute	Chronic	Acute (4–50 dpi)	Chronic (60–730 dpi)
Adipose tissue	ND	Ferreira et al. (21)	Andrade and Silva (76), Buckner et al. (77), Guarner et al. (78), Combs et al. (20)	Guarner et al. (78), Combs et al. (20)
Adrenal gland	ND	ND	Buckner et al. (77), Correa-de-Santana et al. (79)	ND
Blood	Qvarnstrom et al. (80)	Moreira et al. (11), Qvarnstrom et al. (80)	Hoft et al. (69), Buckner et al. (77), Cortez et al. (81), Guillermo et al. (55), Silva et al. (57), Veloso et al. (82), Castro-Sesquen et al. (83)	Veloso et al. (82), Castro-Sesquen et al. (83)
Bone	ND	ND	Morocoima et al. (84)	ND
Bone marrow	Baena Teran et al. (85)	ND	Morocoima et al. (84)	ND
Cartilage	ND	ND	Da Costa et al. (86), Morocoima et al. (84)	ND
Central Nervous System	Mortara et al. (19)	Mortara et al. (19)	Andrade et al. (87), Buckner et al. (77), Guarner et al. (78), Rachid et al. (88)	Andrade et al. (87), Guarner et al. (78)
Gastro Intestinal Tract	Mortara et al. (19)	ND	Andrade et al. (87), Buckner et al. (77), Guarner et al. (78)	Andrade et al. (87), Guarner et al. (78)
Heart	Mortara et al. (19)	Mortara et al. (19), Vago et al. (89), Schijman et al. (90)	Andrade et al. (87), Buckner et al. (77), Guarner et al. (78), Combs et al. (20), Castro-Sesquen et al. (83)	Guarner et al. (78), Combs et al. (20), Castro-Sesquen et al. (83)
Liver	ND	ND	Hoft et al. (69), Buckner et al. (77), Guarner et al. (78)	ND
Lung	Mortara et al. (19)	ND	Guarner et al. (78)	Guarner et al. (78)
Lymph nodes	ND	ND	Hoft et al. (69), Guarner et al. (78), Giddings et al. (74)	Guarner et al. (78)
Muscle	ND	ND	Buckner et al. (77), Guarner et al. (78)	Buckner et al. (77), Guarner et al. (78)
Pancreas	ND	ND	Guarner et al. (78)	Guarner et al. (78)
Peritoneal cells	ND	ND	Silva et al. (57)	ND
Skin	(19)	Mortara et al. (19)	ND	ND
Spleen	ND	ND	Hoft et al. (69), Buckner et al. (77), Guarner et al. (78), Combs et al. (20), Giddings et al. (74)	Guarner et al. (78), Combs et al. (20)
Stomach/esophagus	ND	Vago et al. (91)	Hoft et al. (69), Guarner et al. (78), Cortez et al. (81), Staquicini et al. (92)	Guarner et al. (78)
Thymus	ND	ND	Da Costa et al. (86)	ND
kidney	(19)	ND	Buckner et al. (77), Guarner et al. (78), Castro-Sesquen et al. (83)	Guarner et al. (78), Castro-Sesquen et al. (83)
nasal cavity	ND	ND	Giddings et al. (74)	ND
Bladder	ND	ND	Buckner et al. (77)	Buckner et al. (77)

*ND – not detected*.

In immunocompetent hosts, the high parasitemia observed in the acute phase is quickly controlled through immune effector mechanisms. As a result, *T. cruzi* numbers in the blood and tissues drop drastically to almost undetectable levels as the infected individual enters the chronic phase. Nevertheless, *in situ* PCR (Polymerase Chain Reaction) and confocal analyses have shown that even in the chronic phase, tissues are not parasite-free (11–18). Several tissues, including the heart and the nervous system, as well as adipocytes, retain amastigote forms that perpetuate the chronic infection (19–21). Additionally, Chagas disease may be reactivated during periods of immunosuppression, such as in patients with HIV/AIDS or undergoing immunosuppressive drug therapy (22, 23).

Although controlled, *T. cruzi* persistence in tissues appears to be associated with inflammatory lesions and disease severity in the chronic phase (12, 13, 18, 24–28). Using two models of chronic infection, Zhang and Tarleton (18) demonstrated that parasite clearance from tissues resulted in the disappearance of associated inflammatory lesions and resolution of disease. Taken together, these studies clearly demonstrate that Chagas disease is a systemic infection and that the immune response is important in containing *T. cruzi* replication in the acute phase which impacts disease severity during the chronic phase of the infection.

## Systemic or Mucosal Routes of *T. cruzi* Infection Differentially Affect Parasite Load and Mortality in Mice

Experimental models of *T. cruzi* infection have been widely used to study various aspects of the pathogenesis and pathophysiology of Chagas disease. In fact, the vast majority of our knowledge on the biology of *T. cruzi* infection was initially obtained using experimental mouse models. It is well established that the immune response and immunopathologic manifestations following *T. cruzi* infection are dependent on genetically heterogeneous host populations, parasite strain, inoculum size, and route of infection. Moreover, the anatomical route of pathogen invasion may directly impact upon the host immune response and host resistance (Box 1). In this way, several studies compared mucosal and systemic *T. cruzi* infection and mortality in mice.

Box 1 **T cell differentiation into effector cells following bacterial infections may be differentially programed depending on the route of pathogen presentation (96, 97).**

Accordingly, intravenous infection generates IFN-γ-producing T cells, whereas the mucosal routes of infection (e.g., intranasal or intragastric) generate IL-17-producing T cells. These results are in keeping with the observation that spleen-derived antigen-presenting (APC) cells are prone to producing IL-12, a cytokine involved in Th1 differentiation, whereas mucosal APC rather produce IL-6 and TGF-β, cytokines that selectively drive the Th17 differentiation program (98). In addition, it is interesting to note that these route-driven programs of T cell differentiation may be involved in the fine tuning of innate receptors for bacterial molecules (96) and influence the ability to generate an efficient memory pool of effector cells (97).

In 1967, Marsden showed that CFI mice infected with the *Peruvian* strain of *T. cruzi* by systemic routes [*intraperitoneal (i.p.)*, *intravenous (i.v.)*, or *subcutaneous (s.c.)*] showed higher infection rates (67–100%) and mortality than mucosal routes [*oral (o.i.), intragastric (i.g.), intrarectal (i.r.), genitalia (gen.)*, *or conjunctival (cnj.)*] (17–67%) (29). Similar results were observed in a study by Camandaroba et al. (30) in which *i.p.* and *i.g.* inoculation with the *Peruvian* and *Colombian* strains of *T. cruzi* were compared in Swiss mice. Caradonna and Pereiraperrin (31) infected BALB/c and C57BL/6 mice with the *Tulahuén* strain of *T. cruzi* via *s.c.* and intranasal *(i.n.)* routes and observed higher mortality in the *s.c.* group. Interestingly, mice infected via the *i.n.* route developed higher brain parasitism and lower blood parasitemia than animals infected via the *s.c.* route, suggesting a preferential homing of the parasite to the brain after *i.n*. administration (31).

Taken together, these observations suggest that the route of parasite entry into the host is a key factor in Chagas pathogenesis. It is logical to think that following parasite entry, the initial target tissues/cells in the circulation (Table 2) may contribute to the development of an immune response able to control infection.

**Table 2:** **Primary distribution of *T. cruzi* in infected mice**.

Route of infection	First target tissues/cells	Reference
Intravenous	Blood, liver, spleen, lungs, and kidneys/macrophages	Kuhn et al. (93)
Intraperitoneal	Peritoneal macrophages	Nogueira et al. (94)
Oral	Stomach (epithelial cells), drainage lymph node	Hoft et al. (69), Yoshida (95)
Conjunctival	Nasolacrimal ducts and nasal cavities (epithelial cells), parotid, and submandibular lymph node	Giddings et al. (74)

## Systemic *Trypanosoma cruzi* Entry Induces a Differential Response in Secondary Lymphoid Organs of Infected Mice

Acute and chronic *T. cruzi* infections promote significant increases in the size and numbers of cells in the subcutaneous lymph nodes (SCLN) and spleen (SP), likely due to persistent T and B cell polyclonal activation in these tissues (32, 33). Parasite-derived proteins, such as trans-sialidase and racemase, as well as *T. cruzi*-derived DNA have been shown to contribute to lymphocyte proliferation in Chagas disease (34–36). Interestingly, the majority of polyclonal lymphocytes activated during early *T. cruzi* infection do not appear to be parasite-specific (37–45). However, the relative role of T and B cells in controlling *T. cruzi* infection remains controversial. Although there are data showing that T and B cell activation is necessary for limiting *T. cruzi* expansion, the polyclonal activation also appears to contribute to the pathological alterations observed in Chagas disease (44, 46, 47). Similarly to splenocytes, SCLN-derived effector T cells from infected mice secrete high amounts of IL-2, IL-4, IL-10, and IFN-γ, suggesting the existence of a mixed type-1 and type-2 profiles of cytokine secretion (48, 49).

The process of expansion and contraction by the lymphocyte population in the secondary lymphoid organs can be regarded as a regional response to systemic *T. cruzi* infection. Gut-associated lymphoid tissues are specialized for draining antigens present in the gastrointestinal tract and are also involved in the tolerogenic immune response. In this respect, gut-associated lymphoid tissues may be involved in the progressive damage of the digestive system (megacolon and megaesophagus) that is a consequence of chronic *T. cruzi* infection (50–52). For these reasons, the mesenteric lymph nodes (MLN) and Peyer’s Patches (PP) are also studied in systemic infections, since they may be involved in the gut pathological changes that are observed in infected patients. In contrast to the hyperplasia of the SP and SCLN observed in infected mice, there is a reduction in the size and cell number of the MLN and PP, possibly due to the increased depletion of T and B lymphocytes (49, 53, 54).

As a consequence of cell activation, T lymphocyte apoptosis is also observed in lymphoid tissues (Box 2). In fact, the Fas molecule is one candidate to regulate T and B lymphocyte responses in both the SP and SCLN during the acute infection (33, 54, 55). For example, it has been shown that Fas selectively kills activated IgG^+^ B lymphocytes with specificities for parasite antigens (56). Moreover, SP-derived CD4^+^ and CD8^+^ T cells respond to Fas-induced apoptosis, as they demonstrate increased Fas/FasL expression and caspase-8 activation during acute infection (48, 55). In agreement with these data, it has been shown that the *in vivo* injection of anti-FasL and a general caspase inhibitor (zVAD-fmk) into acutely infected mice impairs T and B lymphocyte death and improves the host immune response to infection in both SCLN and SP (55, 57). Blockade of activated CD8^+^ T cell death increases IFN-γ secretion by splenocytes in the initial stages of infection, and IL-4 and IL-10 are induced at later stages (55, 58).

Box 2 **T cell apoptosis can be stimulated in secondary lymphoid organs by features such as activation-induced cell death (AICD), granzymes, or growth factor withdrawal (59, 60).**

The abundance of antigens and cytokine production (IL-2) in the microenvironment is essential to trigger the cell death pathway (60). In the presence of a given antigen, IL-2 prompt T cells to die by AICD, through activation of death receptor molecules (Fas or TNF-R and caspase-8 activation) (61–63). The absence of antigen, deprivation of cytokines or cytotoxic factors (such as oxidative stress and glucocorticoids) initiates the intrinsic apoptotic pathway, regulated by anti-apoptotic BCL-2 family members that can be divided into three subgroups of proteins: (1) the pro-survival members (Bcl-2, Bcl-x_L_, Mcl-1, A1/Bfl-1, and Bcl-w); (2) the pro-apoptotic BH3-only proteins (Bim, Bid, Puma, Bad, Bmf, Hrk, Bik, and Noxa) activated transcriptionally, post-transcriptionally, or post-translationally by cytotoxic factors; and (3) multi-BH domain pro-apoptotic protein (Bak and Bax) (64–67). The intrinsic pathway of death involves mitochondrial membrane permeabilization, cytochrome c release into the cytoplasm, activation of caspase-9, and downstream effector caspases (61). Interestingly, regulatory T cells deprives the effector T cells of growth factors (such as IL-2), which causes either proliferation arrest and apoptosis mediated by growth factor withdrawal (59, 68).

Diminished numbers of MLN and PP lymphocytes from *T. cruzi*-infected mice appear to be associated with differences in lymphocyte activation, proliferation, and apoptosis. MLN-derived cells from infected mice show reduced numbers of proliferating lymphocytes *in vivo* and decreased cytokine production (IL-2, IL-4, IL-10) *in vitro* by activated T cells, which have been demonstrated to produce mainly type-1 cytokines (33, 49). In addition to Fas, TNFR1/p55-mediated signaling and IL-4 deprivation through caspase-9 activation are involved in T cell death and consequent MLN atrophy seen in the course of acute infection (33, 49). These data suggest that distinct mechanisms are involved in lymphocyte contraction events.

These studies demonstrated that lymphocyte apoptosis in secondary lymphoid organs represents an important feature of the immune response to a given pathogen. In agreement, the *in vivo* administration of zVAD-fmk reduces lymphocyte apoptosis in secondary lymphoid tissues and increases host resistance to *T. cruzi* infection (49). Moreover, SP or MLN cells are involved in the host immune response, as splenectomy or MLN excision prior to *T. cruzi* infection in mice increases susceptibility to infection with elevated blood parasitemia (49, 54). In this context, further studies are necessary to approach apoptosis-associated molecules that might be operating as a consequence of regional response following T cell activation and regulation in the course of *T. cruzi* infection (Box 2).

## Does a Distinct Route of Infection Interfere with Secondary Lymphoid Organ dynamics?

Previous data have revealed that oral *T. cruzi* inoculation results in blood parasitemia and heart tissue parasitism, thereby clearly indicating a systemic infection (30, 69, 70). A primary infection with insect-derived infective forms delivered orally resulted in parasite replication within epithelial cells of the gastric mucosa (69). This initial invasion is related to establishment of a progressive gastritis and allows further systemic dissemination of the parasite. Nonetheless, the short replication period at this mucosal site induces specific immunity, as protection was observed after a secondary mucosal challenge. Such protection apparently involves the specific production of IgA and IgG (69) and possibly employs CCR5-CCL5 signaling (71).

Protection may also be due to IFN-γ-producing lymphocytes as indicated by their increased frequency in the gastric mucosa and draining lymph nodes of orally infected mice (69). Moreover, a mucosal vaccination approach leading to polarized type-1 or type-2 responses (72) as well as the mucosal challenge of genetically deficient mice (73) reinforced the central role for a type-1 response in providing protection following mucosal infection. Interestingly, these humoral and cellular responses are also protective after parasite inoculation in the conjunctival mucosa, a natural portal of entry for *T. cruzi* that leads to nasal infection with subsequent systemic spreading (74).

Following outbreaks of oral contamination by *T. cruzi*, a clear increase in the severity of clinical manifestations was observed in infected patients compared with other types of transmission routes (7, 75). These observations raise important questions concerning the particular features of *T. cruzi* entry via the mucosa, including the possible modulation of local immune mechanisms and the impact on regional and systemic immunity. In fact, we have previously shown that mice infected via both the *i.p.* and *s.c*. routes show similar parasitemia and induce SCLN expansion as well as MLN atrophy (33). Interestingly, the *s.c.* route induced higher SCLN cell expansion and similar MLN atrophy at the peak of parasitemia when compared with the *i.p.* route. These data suggest that unlike SCLN cells, MLN lymphocytes are similarly affected upon infection using both inoculation routes (33).

One can argue that an oral or intragastric infection might impact more severely on the mucosal associated lymphoid organs than the SCLN and SP. We still lack information regarding SCLN behavior in oral infection, but hyperplasia of the lymphoid follicles in the SP has been reported (30). Additionally, Hoft et al. (69) showed that after oral *T. cruzi* infection, BALB/c mice had an increase in gastric lymph node size. In this study, the analysis of cytokine production by gastric lymph node cells and splenocytes showed that IFN-γ and IL-4 were produced in these tissues. These data indicate that in both systemic and mucosal infections splenocytes exhibit a mixed type-1 and type-2 profile of cytokine secretion (48, 49, 69). Regarding the MLN response upon oral infection, no data have been reported in the literature. Therefore, a comparative analysis of oral infections versus other infection routes should be critically performed to better understand the immune mechanisms that are involved in the response to mucosal *T. cruzi* infection.

## Conclusion and Perspectives

Chagas disease is characterized by both protective and immunopathogenic responses. An antigenic challenge in the host elicits a complex protective immune response that includes both inflammatory and regulatory networks. These networks are observed after *T. cruzi* infection and are induced due to systemic infection. However, different routes of parasite entry may impact these immune circuits, define particular regional immune responses, and perhaps change the existing view of how the host mounts a protective immune response. Thus, the current microepidemic of the oral transmission of Chagas disease prompts revisiting previous findings (99). More importantly, new studies investigating the influence of a primary infection with the parasite through mucose should be performed.

## Conflict of Interest Statement

The authors declare that the research was conducted in the absence of any commercial or financial relationships that could be construed as a potential conflict of interest.
